# Genetic Divergence and Heritability of 42 Coloured Upland Rice Genotypes (*Oryzasativa*) as Revealed by Microsatellites Marker and Agro-Morphological Traits

**DOI:** 10.1371/journal.pone.0138246

**Published:** 2015-09-22

**Authors:** Faiz Ahmad, Mohamed Musa Hanafi, Md Abdul Hakim, Mohd Y. Rafii, Ibrahim Wasiu Arolu, Siti Nor Akmar Abdullah

**Affiliations:** 1 Laboratory of Food Crops, Institute of Tropical Agriculture, Universiti Putra Malaysia, 43400, Serdang, Selangor, Malaysia; 2 Laboratory of Plantation Crops, Institute of Tropical Agriculture, Universiti Putra Malaysia, 43400, Serdang, Selangor, Malaysia; 3 Department of Land Management, Faculty of Agriculture, Universiti Putra Malaysia, 43400, Serdang, Selangor, Malaysia; 4 Malaysia Nuclear Agency, 43000, Kajang, Selangor, Malaysia; 5 Department of Agricultural Chemistry, Hajee Mohammad Danesh Science & Technology University, Dinajpur, Bangladesh; CSIRO, AUSTRALIA

## Abstract

Coloured rice genotypes have greater nutritious value and consumer demand for these varieties is now greater than ever. The documentation of these genotypes is important for the improvement of the rice plant. In this study, 42 coloured rice genotypes were selected for determination of their genetic divergence using 25 simple sequence repeat (SSR) primers and 15 agro-morphological traits. Twenty-one out of the 25 SSR primers showed distinct, reproducible polymorphism. A dendrogram constructed using the SSR primers clustered the 42 coloured rice genotypes into 7 groups. Further, principle component analysis showed 75.28% of total variations were explained by the first—three components. All agro-morphological traits showed significant difference at the (p≤0.05) and (p≤0.01) levels. From the dendrogram constructed using the agro-morphological traits, all the genotypes were clustered into four distinct groups. Pearson’s correlation coefficient showed that among the 15 agro-morphological traits, the yield contributing factor had positive correlation with the number of tillers, number of panicles, and panicle length. The heritability of the 15 traits ranged from 17.68 to 99.69%. Yield per plant and harvest index showed the highest value for both heritability and genetic advance. The information on the molecular and agro-morphological traits can be used in rice breeding programmes to improve nutritional value and produce higher yields.

## Introduction

Rice (*Oryza sativa* L.) is the staple food and most important crop in most Asian countries. It belongs to the *Poaceae* family and is the main source of carbohydrate in these countries. Additionally, rice comprises about 20% of calories consumed worldwide [1]. About 90% of both rice production and consumption is from Asian countries and it comprises 80% world rice production and consumption [2]. The demand for rice will reach 800 million tons by 2025 [3]. Upland rice can grow in non-flooded soil. Rice may have special characteristics, such as fragrance, colour and shape [4]. Many varieties have been grown all over the world including white and coloured rice. Coloured rice refers to the genotypes that have a coloured bran layer. It may be red or black, which are referred to as “red” or “black” rice. Genetic factors cause the different bran layer among the rice genotypes [5].

Currently, demand for highly nutritious and healthier food is the norm as people today are more concerned about maintaining a healthy lifestyle. In this regard, coloured rice genotypes have higher content of antioxidant compounds, such as polyphenols, tocochromanols and oryzanols, which have been shown to have a significant effect on human health. Furthermore, these genotypes also contain high micronutrient content, such as iron and zinc [6]. It has been reported that black rice has higher vitamin, mineral and protein content as compared to non-coloured rice [7]. Consequently, the assessment of coloured rice genotypes is very important to determine improvement potential through breeding programmes.

Molecular markers are powerful tools assessment of genetic diversity among plants of the same or different species. Precise characterization of plant genetic diversity is also useful for the development of new genotypes of good quality for breeding programmes [8]. The development of molecular marker provides new opportunity for genetic improvement of rice grain quality [9, 10]. Simple Sequence Repeat (SSR) is widely used in genetic diversity studies because of the high polymorphic nature and accuracy [11].

The agro morphological marker is a traditional tool based on morphological and physiological characteristics. It is inexpensive but not as reliable as the molecular marker as it can be influenced by environmental conditions. This can be seen, where plants of the same species have varied morphological traits due to environmental interaction [12]. Rice genotypes have frequently been classified based on leaf blade, width, leaf colour, colour of auricle and ligule [13]. The combination of morphological and molecular markers provide comprehensive tools for genetic dissection and yield evaluation which is required for selection of genotypes for breeding programmes and for genotype documentation.

Heritability can be used to estimate the structures of a population by determining which characters will be transmitted to the next generation [14]. Breeders use the yield component factor to predict the yield of plants. The yield component factor was found to be the most effective in increasing grain yield whereby the component is highly heritable, genetically independent, and positively correlated with each other [15]. Genetic advance is another criterion that needs to be considered when selecting the traits related to yield contribution. The prediction of genetic advance is important for crop improvement in breeding programmes [16].

Hence the objectives of this study were: i) to estimate the genetic divergence of the 42 selected coloured upland rice genotypes using 25 SSR markers and 15 agro-morphological traits; ii) to calculate the heritability and genetic advance among the 15 traits; iii) To determine the correlation among 15 agro-morphological traits and iv) to identify the genotypes that can potentially be used in breeding programmes.

## Materials and Methods

### Plant materials

Forty two coloured upland rice genotypes were obtained from the International Rice Research Institute (IRRI), Philippines out of which five had purple seed coats while the others were red. The genotypes were selected based on their locality and the colour of the seed coat (Table 1).

**Table 1:** List of selected coloured upland rice genotypes.

Genotypes no	Seed coat colour	Germplasm name	IRGC accession	Country Origin	Status
**v1**	Red	Black banni	10181	India	Landrace/Traditional cultivar
**v2**	Red	258	14887	Liberia	Breeding and inbred line
**v3**	Purple	Khao gam(niaw)	15005	Thailand	Landrace/Traditional cultivar
**v4**	Red	Bi-e-gaw	15053	Thailand	Landrace/Traditional cultivar
**v5**	Red	C	15165	Ivory Coast	Released/Improved/advanced cultivar
**v6**	Red	Choke tang	24085	Vietnam	-
**v7**	Red	Chokoto 14	25988	Brazil	-
**v8**	Red	Ja hau	27654	Thailand	Landrace/Traditional cultivar
**v9**	Red	Ja la shau	27655	Thailand	Landrace/Traditional cultivar
**v10**	Red	Ja loy	27656	Thailand	Landrace/Traditional cultivar
**v11**	Red	Ja no naq	27666	Thailand	Landrace/Traditional cultivar
**v12**	Purple	Ja nu ne ne	27671	Thailand	Landrace/Traditional cultivar
**v13**	Red	Bibili al	31369	Sri Lanka	Landrace/Traditional cultivar
**v14**	Purple	Ngacheik	33453	Myanmar	Breeding and inbred line
**v15**	Red	IR 9669-22-2-6	40315	Philippines	Breeding and inbred line
**v16**	Red	IR 9669-23-12-7	40316	Philippines	Breeding and inbred line
**v17**	Red	IR 9669-PP 823–1	40317	Philippines	Breeding and inbred line
**v18**	Red	IR 9669-PP 830–1	40319	Philippines	Breeding and inbred line
**v19**	Red	IR 9669-PP 836–1	40320	Philippines	Breeding and inbred line
**v20**	Red	IR 5533-13-1-1	40425	Philippines	Breeding and inbred line
**v21**	Red	IR 5533-14-1-1	40426	Philippines	Breeding and inbred line
**v22**	Red	IR 5533-15-1-1	40427	Philippines	Breeding and inbred line
**v23**	Red	IR 5533-50-1-10	40428	Philippines	Breeding and inbred line
**v24**	Red	IR 5533-55-1-11	40429	Philippines	Breeding and inbred line
**v25**	Red	IR 5533-56-1-12	40430	Philippines	Breeding and inbred line
**v26**	Red	IR 5533-PP 854–1	40432	Philippines	Breeding and inbred line
**v27**	Red	IR 5533-PP 856–1	40434	Philippines	Breeding and inbred line
**v28**	Red	IR 9559-3-1-1	40437	Philippines	Breeding and inbred line
**v29**	Red	IR 9559-4-1-1	40440	Philippines	Breeding and inbred line
**v30**	Red	IR 9559-5-3-2	40441	Philippines	Breeding and inbred line
**v31**	Red	IR 9559-PP 871–1	40446	Philippines	Breeding and inbred line
**v32**	Red	IR 3257-13-56	40497	Philippines	Breeding and inbred line
**v33**	Red	Chirikata 2	66264	India	-
**v34**	Red	Ippa	67833	Bhutan	Landrace/Traditional cultivar
**v35**	Purple	Beu e-soo	73363	Thailand	-
**v36**	Purple	Daeng se leuad	73403	Thailand	-
**v37**	Red	Chirikata 1	74580	India	-
**v38**	Red	Biaw bood pae	76318	Thailand	-
**v39**	Red	Blau noc	90567	Vietnam	Landrace/Traditional cultivar
**v40**	Red	Ble chu cau	90579	Vietnam	Landrace/Traditional cultivar
**v41**	Red	Ble la	90584	Vietnam	Landrace/Traditional cultivar
**v42**	Red	Ble lia su	90587	Vietnam	Landrace/Traditional cultivar

### Experimental design and layout

The selected forty two coloured rice genotypes were germinated in a petri dish after which they were transferred into growing buckets (23× 21 cm) in the glasshouse at the rate of 5 plants/bucket. The experimental design was randomized complete block design (RCBD) with three replications.

Agronomic practice such as weed control was done manually while disease control was through the application of 5g per bucket Furadan (PT Bina Guna Kimia, Indonesia) and 5 ml per 1 L Malathion (Hextar chemicals Sdn. Bhd., Malaysia). The fertilisers urea, muriate of potash (MOP), and triple super phosphate (TSP) were applied 3 times at 5, 25, and 55 days after planting, to provide N, K and P nutrition, at the rate of 160 kg N/ha, 80 kgP_2_O_5_/ha, and 60 kgK_2_O/ha.

### Data collection

Fifteen agro-morphological traits were identified by measuring five plants per genotype in each replicate and their means were used for further analysis. These traits include: (i) plant height; (ii) number of tillers per plant; (iii) number of panicles per plant; (iv) percentage of filled grain; (v) 100 grain weight; (vi) harvest index; (vii) days to first flowering; (viii) days to maturity; (ix) grain dimension and shape; (iix) length of flag leaf; (xi) panicle length; (xii) kernel length; (xiii) length breadth ratio;and (xiv) chlorophyll SPAD reading at 40 days and (xv) 60 days.

### DNA extraction protocol

DNA was extracted from the seed samples using the modified conventional method [17]. About 100 mg of each seed sample was ground in the mortar using a pestle, and 400 μL extraction buffer (200 mMTris-HCL, 200 mMNacl, 25mM EDTA, 0.5% SDS). Then, the solution was transferred into a 2 ml microcentrifuge tube and 400 μl of CTAB solution (2% CTAB, 100 mMTris-HCL, 20 mM EDTA, 1.4 M NaCl, 1% PVP) was added. Next, 400 μl mixture, ratio of chloroform: isoamyl alcohol: phenol (24:1:5%), was added in the same tube. The mixtures were then well mixed by vortex and centrifuge (14,000 rpm, 5 minutes) at room temperature. The supernatant was then transferred into a new 2 mL microcentrifuge tube and 2/3 volume of isopropanol added. The mixtures were gently mixed by inverting the microcentrifuge tube and then incubated at room temperature for 10 minutes. Following this the mixture was centrifuged again (14,000 rpm, 5 minutes) at room temperature. The supernatant was then discarded and the pellets rinsed with 70% alcohol for a few minutes. Subsequently, pellets were air dried and re-suspended in 50 μL of TE buffer. The quality and quantity of the DNA was measured by NanoDrop ND-1000 spectrophotometer (Thermo Fisher Scientific, USA).

### SSR-PCR analysis

The DNA extracted from the seeds were genotyped using 25 SSR rice primers obtained from Gramene (http://www.gramene.org), as shown in Table 2. Each PCR reaction total of 25 uL contained 5x PCR buffer (5 uL), MgCl_2_ (3 uL), dNTPs (0.5 uL), Taq polymerase (0.2 uL), water (14.9 uL), DNA template (1 uL), and Primer (0.2 uL). The PCR was performed using a Biometra thermo cycler with the following program: initial denaturation (95°C, 5 min), 35 cycles of denaturation (94°C, 1 min), annealing (depending on the primer, 1 min), extension (72°C, 5 min), and finally, final extension (72°C, 5 min). The PCR products were resolved using 3% metaphor agarose gel at 75V for 65 minutes with ethidium bromide as a stain. Following this the gel was viewed under UV transilluminator.

**Table 2:** List of information SSR primers used in this study.

Marker	Forward Primer	Reverse Primer	Anneal temp	Min Allele	Max Allele
**RM495**	**aatccaaggtgcagagatgg**	**caacgatgacgaacacaacc**	**55**	**148**	**160**
**RM283**	**gtctacatgtacccttgttggg**	**cggcatgagagtctgtgatg**	**61**	**130**	**176**
**RM259**	**tggagtttgagaggaggg**	**cttgttgcatggtgccatgt**	**55**	**133**	**186**
**RM312**	**gtatgcatatttgataagag**	**aagtcaccgagtttaccttc**	**55**	**86**	**106**
**RM431**	**tcctgcgaactgaagagttg**	**agagcaaaaccctggttcac**	**55**	**233**	**261**
**OSR13**	**catttgtgcgtcacggagta**	**agccacagcgcccatctctc**	**53**	**85**	**122**
**RM338**	**cacaggagcaggagaagagc**	**ggcaaaccgatcactcagtc**	**55**	**178**	**184**
**RM514**	**agattgatctcccattcccc**	**cacgagcatattactagtgg**	**55**	**229**	**278**
**RM413**	**ggcgattcttggatgaagag**	**tccccaccaatcttgtcttc**	**53**	**71**	**114**
**RM178**	**tcgcgtgaaagataagcggcgc**	**gatcaccgttccctccgcctgc**	**69**	**112**	**131**
**RM334**	**gttcagtgttcagtgccacc**	**gactttgatctttggtggacg**	**55**	**119**	**207**
**RM133**	**ttggattgttttgctggctcgc**	**ggaacacggggtcggaagcgac**	**63**	**226**	**237**
**RM510**	**aaccggattagtttctcgcc**	**tgaggacgacgagcagattc**	**57**	**99**	**127**
**RM455**	**aacaacccaccacctgtctc**	**agaaggaaaagggctcgatc**	**57**	**127**	**144**
**RM118**	**ccaatcggagccaccggagagc**	**cacatcctccagcgacgccgag**	**67**	**149**	**165**
**RM408**	**caacgagctaacttccgtcc**	**actgctacttgggtagctgacc**	**55**	**112**	**128**
**RM152**	**gaaaccaccacacctcaccg**	**ccgtagaccttcttgaagtag**	**53**	**133**	**157**
**RM44**	**acgggcaatccgaacaacc**	**tcgggaaaacctaccctacc**	**53**	**82**	**132**
**RM433**	**tgcgctgaactaaacacagc**	**agacaaacctggccattcac**	**53**	**216**	**248**
**RM447**	**cccttgtgctgtctcctctc**	**acgggcttcttctccttctc**	**55**	**95**	**146**
**RM316**	**ctagttgggcatacgatggc**	**acgcttatatgttacgtcaac**	**55**	**194**	**216**
**RM271**	**tcagatctacaattccatcc**	**tcggtgagacctagagagcc**	**55**	**80**	**120**
**RM171**	**aacgcgaggacacgtacttac**	**acgagatacgtacgcctttg**	**55**	**307**	**347**
**RM287**	**ttccctgttaagagagaaatc**	**gtgtatttggtgaaagcaac**	**55**	**82**	**118**
**RM144**	**tgccctggcgcaaatttgatcc**	**gctagaggagatcagatggtagtgcatg**	**57**	**216**	**295**

(Source: www.gramene.org)

### Data analysis

The presence and absence of alleles were scored using the binary system ‘1’ and ‘0’ respectively. The observed number of alleles, effective number of alleles and Shannon's Information index were determined using the Popgen software. Polymorphism information content (PIC) and expected heterozygosity (He) were also calculated using the PIC calculator (http://www.liv.ac.uk/~kempsj/pic.html).

Genetic similarity was calculated using the dice coefficient[18]; the sequential agglomerative hierarchal and nested (SAHN) clustering was performed based on genetic similarity and unweighted paired group method with arithmetic averages (UPGMA); the principal component analysis (PCA) was performed on the matrix of the genetic similarity coefficients; and data analysis was carried out using the NTSYS version 2.1.

The agro-morphological traits collected were subjected to Analysis of Variance (ANOVA) using SAS version 9.2. The Pearson correlation coefficient among all agro-morphological traits calculated using SAS version 9.2. A dendrogram was then constructed based on UPGMA and PCA based on agro-morphological data using NTSYS version 2.1. The broad sense heritability, genetic advance and other variance estimates were calculated using the method of Allard [19]. The following formulas were used to calculate the genetic parameters:

Genotypic variance (σ²_g_) = (MS_2_ –MS_3_)/b

Error variance (σ²_e_) = MS_3_

Phenotypic variance (σ²_p_) = σ²_g_ + σ²_e_

Genotypic coefficient of variation $\left(\text{GCV}\right)\;=\sqrt{\sigma^{2}\text{g}}\;/\;\bar{\text{X}}\times 100$

Phenotypic coefficient of variation $\left(\text{PCV}\right)\;=\sqrt{\sigma^{2}\text{p}}\;/\;\bar{\text{X}}\times 100$

MS_2_ = mean square of populations

MS_3_ = mean square of error

b = number of blocks

$\bar{\text{X}}$ = mean of the trait

Heritability (h^2^_B_) = σ²_g/_σ²_p_

Expected genetic advance (GA) and genetic gain (GG) (as percentage of the mean) were calculated using the method of Allard [15] where selection intensity (K) was assumed to be 5%

Expected genetic advance $\sqrt{\sigma^{2}\text{p}\;}(\text{GA})=\text{K}\times \sqrt{\sigma^{2}\text{p}}{\times \text{h}}^{2}{}_{\text{B}}$

K is a constant which represents the selection intensity, when K is 5% the value is 2.06, $\sqrt{\sigma^{2}\text{p}}$ represents the phenotypic standard deviation, h_B_^2^ is the heritability while $\bar{\text{X}}$ represents the mean of the characteristic being evaluated, using the formulae suggested by Burton [20].

## Results

### Characterization of SSR markers

**Fig 1:**
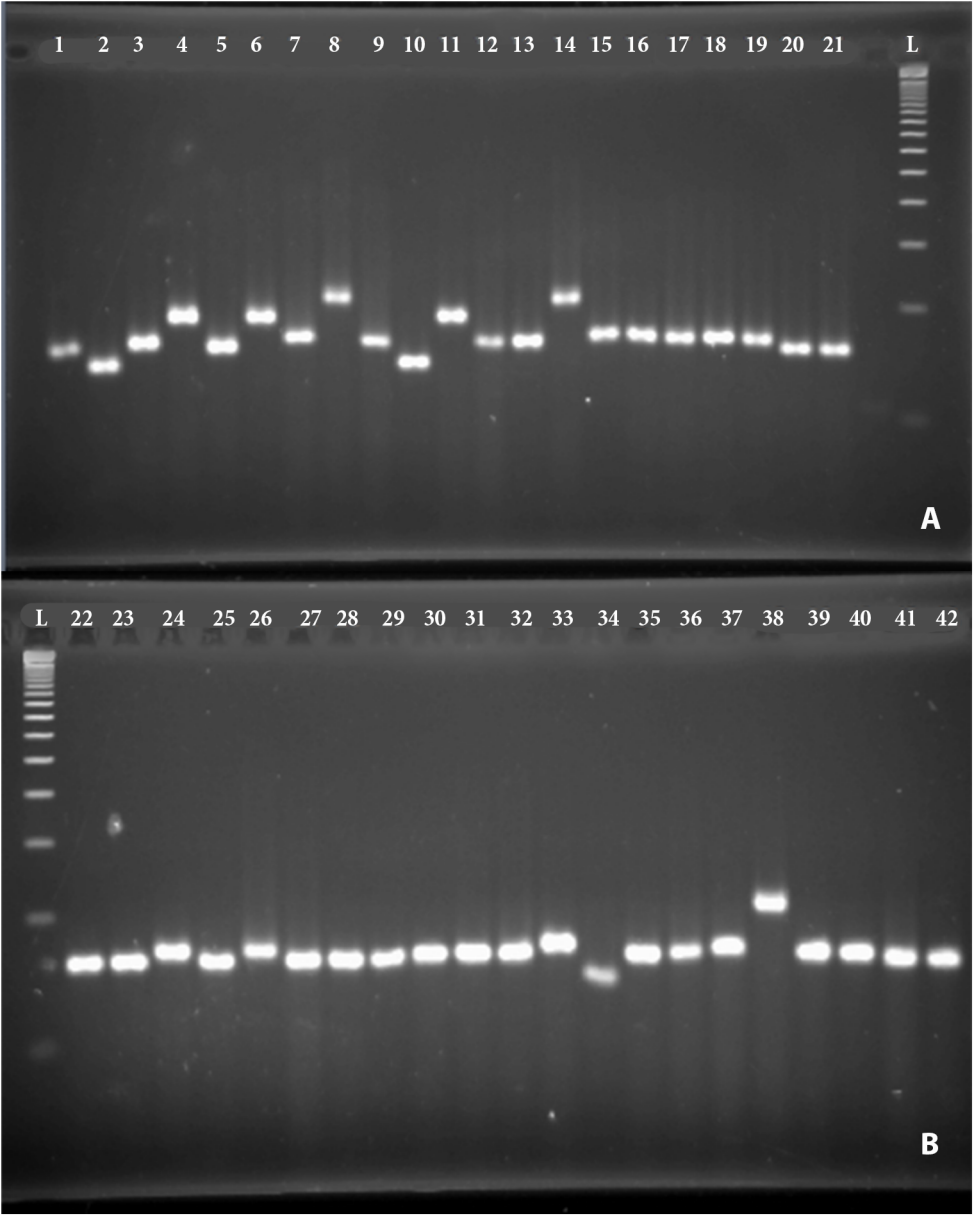
Polymorphic band for marker RM 413 among 42 genotypes.

Out of 25 pairs of SSR primers used for the genotyping of the 42 coloured rice genotypes, 21 showed distinct, clear, and reproducible polymorphism (Fig 1). Only 4 loci were monomorphic amongst the 81 loci (RM 338, RM 431, RM 118 and RM 133). Majority of polymorphic loci had 3 alleles (33.33%). The PIC SSR loci tested ranged from 0.17 (RM 312) to 0.76 (RM 455) with mean of 0.41. Mean of expected heterozygosity (He) was 0.466. The Shannon’s information index (Lewontin, 1972) ranged from 0.38 to 1.35 with the mean of 0.83 (Table 3).

**Table 3:** Expected Heterozygosity (He), Polymorphic Information Content (PIC), and Shannon’s Information Index of 25 SSR markers.

SSR marker	Observed number of allele	Effective number of allele	He	PIC	Shannon's Information index
**RM44**	4	3.184	0.690	0.630	1.240
**RM338**	1	1.000	0.000	0.000	0.000
**RM408**	2	1.960	0.490	0.370	0.680
**RM413**	6	2.960	0.660	0.630	1.380
**RM431**	1	1.000	0.000	0.000	0.000
**RM495**	2	1.634	0.390	0.310	0.580
**RM287**	5	3.514	0.720	0.660	1.350
**RM118**	1	1.000	0.000	0.000	0.000
**RM13**	5	2.471	0.600	0.540	1.120
**RM144**	2	1.999	0.500	0.370	0.690
**RM510**	3	1.661	0.400	0.340	0.650
**RM152**	3	2.477	0.600	0.530	1.000
**RM171**	3	2.252	0.560	0.460	0.880
**RM283**	4	3.142	0.680	0.610	1.190
**RM178**	2	1.616	0.380	0.310	0.570
**RM259**	4	2.129	0.530	0.490	1.020
**RM433**	3	1.852	0.460	0.360	0.700
**RM447**	4	2.882	0.650	0.590	1.140
**RM334**	6	4.333	0.770	0.740	1.610
**RM455**	6	4.794	0.790	0.760	1.660
**RM514**	3	2.662	0.620	0.550	1.040
**RM271**	3	1.648	0.390	0.330	0.650
**RM312**	3	1.225	0.180	0.170	0.380
**RM133**	1	1.000	0.000	0.000	0.000
**RM316**	4	2.459	0.590	0.550	1.100
**Average**	**3.24**	**2.274**	**0.470**	**0.410**	**0.830**

Note: He- Expected heterozygosity, Pic-Polymorphic information content

### Cluster analysis from SSR markers

**Fig 2:**
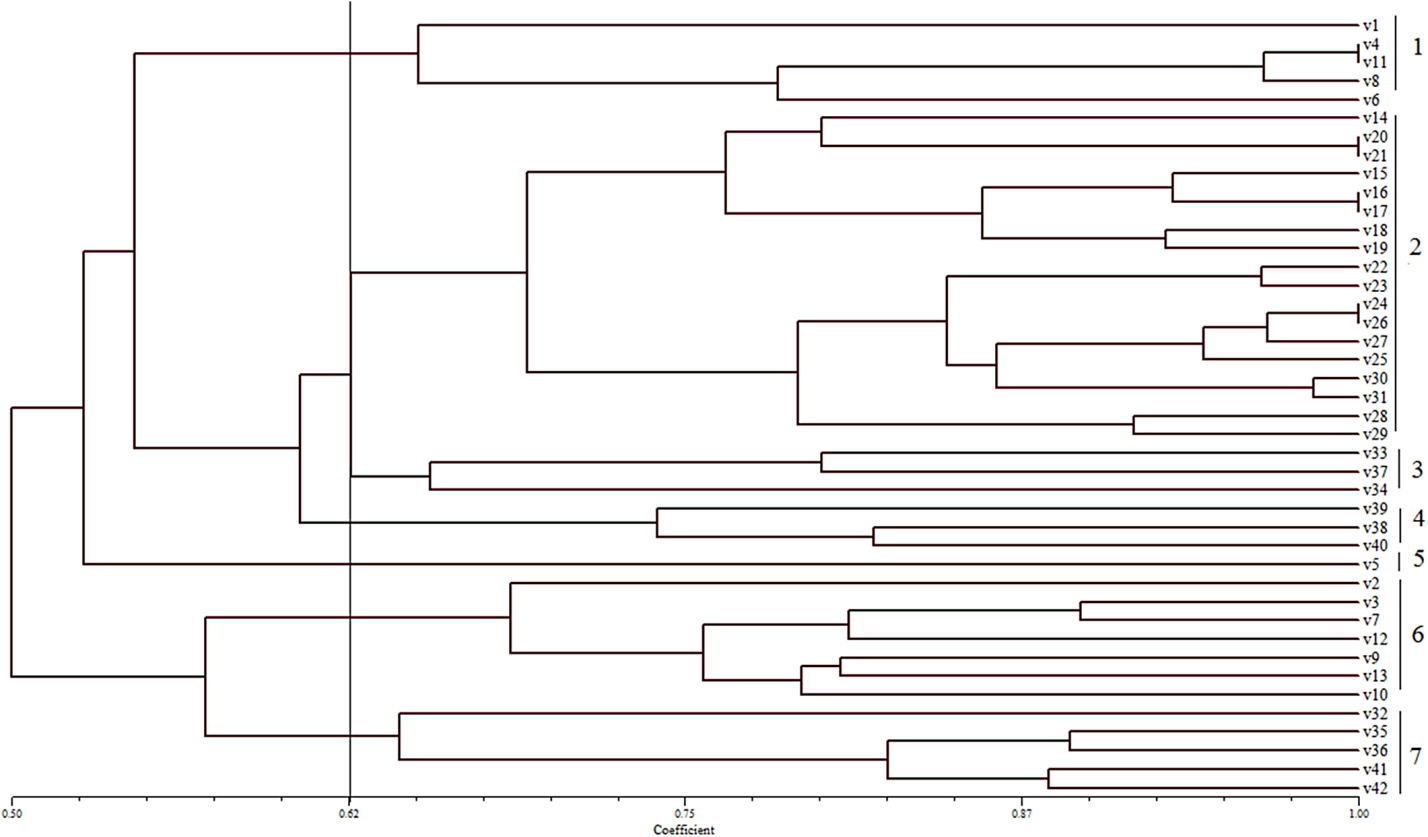
Dendrogram constructed from SSR marker analysis.

The dendrogram based on the UPGMA method grouped the 42 selected coloured upland genotypes into seven groups (Fig 2) at the coefficient of 0.62. Dice coefficient ranged from 0.50 to 1.00. Group 1 comprised the landrace cultivars from Thailand, India and Vietnam. Group 2 comprised all breeding and inbred line genotypes from Philippines and one from Myanmar whereas Group 5 comprised just one genotype from the Ivory Coast.

### Principal component analysis from SSR markers

**Fig 3:**
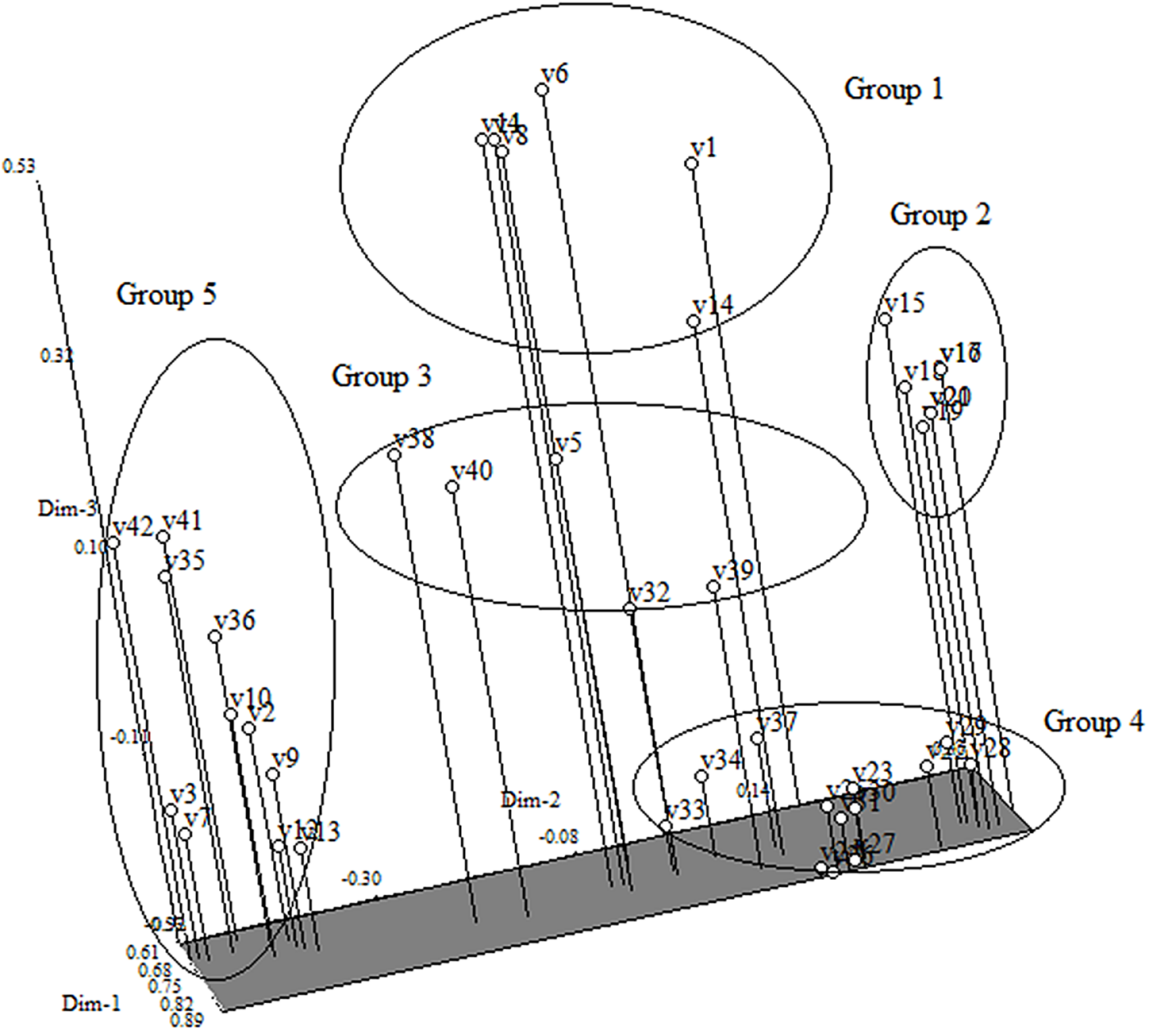
Principle component analysis (PCA) from SSR markers analysis.

The first three components from the PCA analysis explained about 75.28% of the total variation present in these genotypes. About 5 distinct groups were obtained from the three dimensional PCA (Fig 3). Group 1 comprised landraces and traditional cultivars from India, Thailand and Vietnam. The clustering patterns found in these genotypes group via PCA was similar to that found in the dendrogram where all the breeding and inbred lines clustered to form Groups II and group IV, respectively.

### Genetic divergence and morphometric variability in the genotypes

All the genotypes showed higher significant differences at p≤0.05 and p≤0.01 based on the quantitative traits, as revealed by ANOVA (Table 4). Genotype (V22) IR 5533-15-1-1 showed the highest number of tillers and panicles. Plant height ranged from 82.14 to 170.20 cm for all the genotypes with 124.72 cm being the average (Table 5). Genotype V5(C) recorded the highest panicle length at 30.35 cm. while genotype V33 (chirikata 2) showed the highest percentage of filled grain. Additionally, wide variability was observed in grain yield per plant among all the genotypes ranging from 0.57 V13 (Bibili al) to 6.62 g V5 (C) and showed the lowest and highest grain yield per plant. Genotype V7 (Chokoto 14) showed the highest rice kernel length. Length breadth ratio for all genotypes ranged from 2.77 to 4.85, while genotype V4 (Bi-e-gaw) recorded the earliest days for 50% flowering and days to maturity, which was 49 and 114 days, respectively.

**Table 4:** Mean squares of analysis of variance for 15 agro-morphological characteristic among 42 rice genotypes.

Source	Df	NT	NP	PH	LF	PL	%FG	100GW	HI	Y/P	Dflower	Dmature	Klength	Lbratio	Chloro40	Chloro60
**Block**	2	0.05^ns^	0.08^ns^	224.18^ns^	103.88^ns^	0.34^ns^	46.35^ns^	0.002^ns^	0.03^ns^	0.66^ns^	3.31^ns^	4.98^ns^	0.002^ns^	0.0001^ns^	2.82^ns^	13.28^ns^
**Genotypes**	41	0.99**	0.98**	2112.97*	354.61**	65.95**	211.34**	1.47**	0.19**	5.74*	573.26**	744.49**	0.97**	0.45**	18.69*	14.49**
**Error**	82	0.18	0.2	37.17	31.39	2.23	36.29	0.008	0.01	0.56	2.55	1.73	0.001	0.002	11.36	5.53

Note:Df- degree of freedom NT-number of tiller, NP-number of panicle, PH-plant height, LF-length of flag leaf, PL-panicle length, %FG-percentage filled grain, % UFG- percentage unfilled grain, 100GW- 100 grain weight, HI- harvest index, Y/P- yield per plant, Dflower- days to flowering, Dmature- days to maturity, Klength-kernel length, Lbratio-length breadth ratio, Chloro40- chlorophyll SPAD reading at 40 days, Chloro 60- chlorophyll SPAD reading at 60 days

*- significant at 0.05 level

**- significant at 0.01 level, ns- not significant

**Table 5:** Data mean of 15 agro-morphological traits of 42 coloured upland rice genotypes.

Accession name	No tillers	No panicles	Plant height (cm)	Length flag leaf (cm)	% filled grain	100 grain weight (g)	Harvest index	Panicle length (cm)	Length (mm)	L/B ratio	Grain yield/plant (g)	Days first flowering	Days to maturity	Chloro spad 40d	Chloro spad 60d
**Black Banni**	2.42 ± 0.21	2.42± 0.21	162.55± 13.20	62.68± 4.33	64.11± 3.67	2.08±0.01	0.63±0.05	30.19± 0.83	5.4±0.06	3.02±0.01	3.60±0.68	69±1.15	129±0.58	37.27±1.77	38.37±1.08
**258**	2.99± 0.26	2.86± 0.33	134.28± 7.38	43.60± 5.62	58.17± 6.28	2.00±0.03	0.37±0.06	19.18± 0.57	5.38±0.01	3.36±0.01	2.69±0.12	88±1.45	151±1.0	36.73±1.20	37.27±1.49
**Khao Gam(Niaw)**	2.25± 0.25	2.25± 0.25	138.05± 3.93	47.88± 3.13	63.13± 1.90	2.61±0.05	0.73±0.09	27.42± 0.51	5.48±0.01	3.01±0.01	2.97±0.21	100±0.88	171±0.88	37.17±1.47	34.80±2.24
**Bi-E-Gaw**	3.14± 0.27	3.14± 0.27	129.25± 17.01	51.04± 2.37	61.85± 1.69	2.08±0.03	0.98±0.01	18.15± 0.13	5.76±0.01	3.51±0.01	2.52±0.07	49±0.58	114±0.58	41.27±0.43	39.13±0.84
**C**	3.18± 0.01	3.18± 0.01	148.12± 2.32	53.79± 4.50	79.45± 4.41	2.16±0.01	0.93±0.07	30.35± 0.65	6.06±0.01	4.85±0.01	6.62±0.24	66±0.58	126±0.88	33.9±0.96	38.80±3.21
**Choke Tang**	2.5± 0.17	2.5± 0.17	170.05± 8.62	52.26± 1.15	59.96± 1.91	2.75±0.08	0.64±0.04	23.63± 1.37	6.23±0.01	3.40±0.06	4.23±0.37	72±0.88	141±0.58	37.17±0.60	37.83±1.75
**Chokoto 14**	2.45± 0.16	2.25± 0.28	136.6± 3.11	53.45± 3.71	54.67± 2.40	3.21±0.08	0.38±0.03	25.83± 0.67	8.06±0.01	4.38±0.01	2.29±0.16	86±0.88	146±0.58	37.57±2.10	39.50±0.93
**Ja Hau**	2.85± 0.73	2.85± 0.73	138.74± 5.50	54.10± 2.80	67.49± 5.55	2.22±0.03	0.98±0.11	20.16± 2.32	5.7±0.06	3.43±0.01	2.42±0.21	55±1.45	115±0.58	40.10±0.95	42.17±0.64
**Ja La Shau**	2.25± 0.52	2.25± 0.52	147.22± 8.24	54.52± 1.75	57.53± 4.68	2.26±0.05	0.75±0.04	29.41± 1.11	6.68±0.01	4.07±0.01	3.47±0.84	75±0.58	138±0.33	37.40±0.47	38.87±1.92
**Ja Loy**	2.27± 0.39	2.27± 0.39	143.26± 6.40	49.06± 3.89	54.33± 1.16	2.44±0.07	0.68±0.03	25.76± 0.22	6.33±0.01	3.64±0.01	4.25±0.72	71±1.20	136±0.88	39.30±0.35	39.80±1.11
**Ja No Naq**	2.00± 0.03	2.00± 0.03	143.73± 5.31	53.74± 2.25	75.42± 0.74	2.66±0.05	0.97±0.02	21.72± 1.05	6.22±0.01	3.33±0.01	3.56±0.20	59±1.76	127±0.58	40.97±0.23	40.83±1.03
**Ja Nu Ne Ne**	2.50± 0.05	2.00± 0.01	108.50± 0.87	43.30± 0.43	30.48± 0.16	3.38±0.003	0.09±0.003	22.4± 0.16	5.54±0.01	2.77±0.01	0.77±0.02	85±0.88	155±0.88	35.83±0.82	37.00±2.36
**Bibili Al**	1.00± 0.01	1.00± 0.02	100.50± 0.29	22.75± 0.08	34.16± 0.28	2.17±0.01	0.09±0.003	17.00± 0.05	4.86±0.01	2.93±0.01	0.57±0.003	83±1.15	156±0.58	40.67±0.70	33.00±0.7
**Ngacheik**	2.62± 0.03	1.66± 0.01	139.02± 0.57	43.46± 0.18	29.90± 0.37	2.53±0.01	0.10±0.01	24.3± 0.03	6.37±0.01	3.88±0.01	0.60±0.03	122±0.58	184±0.88	35.93±0.57	36.50±0.81
**IR 9669-22-2-6**	2.77± 0.15	2.77± 0.15	109.34± 7.16	44.81± 7.57	70.97±4.85	2.54±0.04	0.73±0.10	22.94± 0.64	5.88±0.01	3.14±0.01	5.64±0.28	90±1.20	150±0.88	39.37±0.62	39.63±1.56
**IR 9669-23-12-7**	3.27± 0.27	3.27± 0.27	97.38± 1.40	32.99± 0.61	64.01±4.13	2.89±0.03	0.66±0.03	21.91± 0.35	6.76±0.01	3.8±0.06	5.38±0.22	88±0.58	147±0.33	40.10±0.95	41.87±0.23
**IR 9669-PP 823–1**	2.09± 0.32	1.98± 0.33	108.43± 2.85	35.16± 1.32	65.47±1.75	2.83±0.05	0.56±0.07	21.53± 1.13	6.30±0.06	3.42±0.01	3.90±0.61	91±0.58	159±1.20	40.60±0.64	43.40±0.35
**IR 9669-PP 830–1**	2.97± 0.22	2.97± 0.22	108.76± 8.34	41.77± 3.71	79.77±3.57	2.35±0.02	0.95±0.11	22.36± 0.50	5.36±0.01	2.96±0.01	5.45±0.62	92±0.33	155±0.58	40.97±0.73	42.03±1.17
**IR 9669-PP 836–1**	3.59± 0.43	3.48± 0.53	116.64± 3.64	48.65± 4.68	61.89±5.58	2.08±0.08	0.67±0.11	25.59± 0.63	5.43±0.01	3.31±0.01	4.79±0.74	89±0.33	154±0.88	40.30±0.79	40.40±1.17
**IR 5533-13-1-1**	3.05± 0.28	3.05± 0.25	82.14± 3.84	28.33± 0.34	73.59±3.07	2.63±0.02	0.78±0.03	17.6± 0.60	5.85±0.01	3.2±0.01	4.21±0.19	92±0.58	156±0.88	38.43±0.44	39.83±0.27
**IR 5533-14-1-1**	3.44± 0.06	3.17± 0.17	82.54± 2.67	31.76± 2.13	79.77±3.63	2.40±0.02	0.68±0.10	19.13± 1.83	6.05±0.01	3.54±0.01	4.07±0.34	94±0.58	156±0.88	40.37±0.79	41.27±1.60
**IR 5533-15-1-1**	3.82± 0.01	3.82± 0.01	87.70± 0.61	28.07± 0.28	69.63±0.79	2.66±0.01	0.69±0.04	17.2± 0.28	6.06±0.01	3.26±0.01	4.35±0.10	94±0.58	153±0.67	41.73±0.32	41.40±0.93
**IR 5533-50-1-10**	3.11± 0.15	3.11± 0.11	86.59± 5.88	24.32± 1.39	68.46±6.24	2.47±0.10	0.56±0.07	16.29± 0.54	6.07±0.01	3.57±0.01	2.73±0.49	94±0.58	160±0.58	38.90±1.17	35.10±0.67
**IR 5533-55-1-11**	1.97± 0.24	1.97± 0.24	112.91± 4.73	35.25± 2.25	55.94±3.90	2.37±0.14	0.24±0.06	19.11± 1.18	6.27±0.01	3.30±0.01	1.36±0.35	97±0.58	154±0.58	37.00±0.23	39.80±1.06
**IR 5533-56-1-12**	3.13± 0.13	2.90± 0.17	113.06± 2.02	42.12± 2.57	71.15±2.17	2.59±0.04	0.68±0.10	23.16± 0.30	6.58±0.01	3.58±0.01	4.28±0.61	94±0.88	159±0.58	38.90±0.15	37.43±1.09
**IR 5533-PP 854–1**	2.50± 0.17	2.43± 0.20	113.43± 4.73	35.96± 1.72	76.49±0.44	2.72±0.01	0.52±0.05	22.06± 0.33	6.33±0.01	3.26±0.01	2.76±0.09	99±0.58	165±0.58	37.40±0.76	36.93±0.23
**IR 5533-PP 856–1**	3.43± 0.19	3.37± 0.13	112.54± 0.68	40.49± 10.65	76.41±2.95	2.60±0.07	0.77±0.03	23.14± 0.41	6.37±0.01	3.66±0.01	5.15±0.43	93±0.58	153±0.33	38.60±0.3	39.60±1.04
**IR 9559-3-1-1**	3.11± 0.1	3.05± 0.30	93.53± 2.70	29.36± 2.39	66.25±3.05	2.57±0.04	0.63±0.03	18.41± 0.23	5.71±0.01	3.12±0.01	3.62±0.17	100±0.58	163±0.58	40.30±0.29	39.60±0.2
**IR 9559-4-1-1**	2.51± 0.15	2.51± 0.15	104.61± 7.58	37.5± 2.44	69.36±2.48	2.62±0.05	0.96±0.02	20.8± 0.60	5.74±0.01	3.21±0.01	4.31±0.25	86±0.58	146±0.67	40.87±0.71	39.57±1.56
**IR 9559-5-3-2**	3.44± 0.20	3.22± 0.34	91.56± 3.28	33.08± 2.13	71.21±2.02	2.67±0.03	0.58±0.06	18.06± 0.81	6.11±0.01	3.36±0.01	3.61±0.53	90±1.73	150±0.58	39.17±0.52	38.40±0.32
**IR 9559-PP 871–1**	3.65± 0.35	3.65± 0.35	87.16± 3.03	28.83± 1.74	67.67±3.50	2.42±0.08	0.49±0.05	16.35± 0.25	5.85±0.01	3.31±0.01	2.87±0.11	88±1.15	150±0.88	39.97±0.58	35.70±2.45
**IR 3257-13-56**	2.89± 0.22	2.44± 0.11	93.66± 7.01	27.67± 2.99	74.88±3.51	1.57±0.03	0.28±0.04	15.13± 1.40	4.54±0.01	3.17±0.01	1.53±0.05	90±1.15	153±1.20	39.23±1.11	40.87±1.54
**Chirikata 2**	2.80± 0.01	2.90± 0.06	143.39± 11.69	47.52± 2.99	82.90±1.46	2.84±0.03	0.96±0.07	22.81± 0.50	6.02±0.01	3.20±0.01	4.84±0.31	66±0.58	131±0.33	33.63±1.27	38.40±1.80
**Ippa**	2.43± 0.09	2.43± 0.09	170.2± 13.05	63.7± 3.27	70.02±1.22	1.97±0.05	0.57±0.03	28.51± 0.87	5.98±0.01	3.59±0.01	4.21±0.18	80±0.58	147±1.45	37.20±0.72	38.70±0.80
**Beu E-Soo**	1.96± 0.22	1.96± 0.22	117.98± 0.40	50.25± 1.91	52.46±3.08	2.28±0.06	0.41±0.08	24.25± 1.29	6.12±0.01	3.80±0.06	2.34±0.52	80±0.88	143±0.88	37.33±1.98	41.00±1.39
**Daeng Se Leuad**	2.07± 0.07	2.07± 0.07	162.54± 5.04	48.26± 3.87	60.13±4.93	3.11±0.02	0.94±0.14	29.38± 0.89	6.02±0.01	2.97±0.01	4.57±0.85	64±0.88	127±0.88	39.47±0.87	41.03±1.68
**Chirikata 1**	2.08± 0.12	2.31± 0.06	157.02± 6.50	56.76± 1.16	81.59±5.72	2.46±0.03	0.91±0.08	22.33± 0.54	5.80±0.06	3.37±0.01	3.90±0.31	69±0.88	132±0.88	35.13±0.43	40.00±1.31
**Biaw Bood Pae**	2.20± 0.12	2.20± 0.12	151.67± 8.11	49.47± 2.36	53.75±0.72	2.62±0.05	0.82±0.06	22.42± 0.08	6.41±0.01	3.50±0.01	2.28±0.10	56±0.88	120±0.88	37.77±1.02	35.73±0.96
**Blau Noc**	2.16± 0.26	2.16± 0.26	170.19± 2.60	53.63± 4.77	75.51±1.80	2.29±0.08	0.81±0.08	23.99± 1.33	5.47±0.01	3.14±0.01	3.54±0.42	67±1.15	129±0.58	37.10±0.64	40.53±2.33
**Ble Chu Cau**	2.47± 0.37	2.47± 0.37	141.66± 6.76	46.61± 1.78	78.35±3.15	2.52±0.06	0.97±0.01	23.63± 0.76	5.88±0.01	3.28±0.01	5.13±0.93	56±1.15	120±0.58	36.70±0.75	37.47±1.01
**Ble La**	2.36± 0.10	2.36± 0.10	138.18± 5.42	53.31± 1.01	63.83±4.86	2.62±0.02	0.81±0.05	27.87± 1.07	6.19±0.01	3.64±0.01	3.41±0.32	69±0.58	132±0.58	35.70±2.15	39.87±0.84
**Ble Lia Su**	2.60± 0.14	2.60± 0.14	143.66± 7.20	59.3± 1.28	55.88±6.22	2.98±0.06	0.85±0.10	29.07± 0.38	6.75±0.01	3.44±0.01	4.05±0.70	68±1.20	132±1.20	39.83±0.93	38.60±1.17
**Mean**	**2.67**	**2.6**	**124.72**	**43.82**	**64.95**	**2.51**	**0.66**	**22.63**	**6**	**3.42**	**3.54**	**81.33**	**144.88**	**38.41**	**39**
**Std deviation**	**0.67**	**0.68**	**26.85**	**11.77**	**9.69**	**0.35**	**0.27**	**4.81**	**0.57**	**0.38**	**1.5**	**13.78**	**15.67**	**3.69**	**2.93**
**CV (%)**	**24.91**	**26.06**	**21.53**	**26.88**	**14.33**	**14.11**	**40.48**	**20.59**	**9.44**	**11.21**	**42.39**	**17.08**	**10.79**	**9.67**	**7.51**

### Clustering analysis from agro-morphological traits data

**Fig 4:**
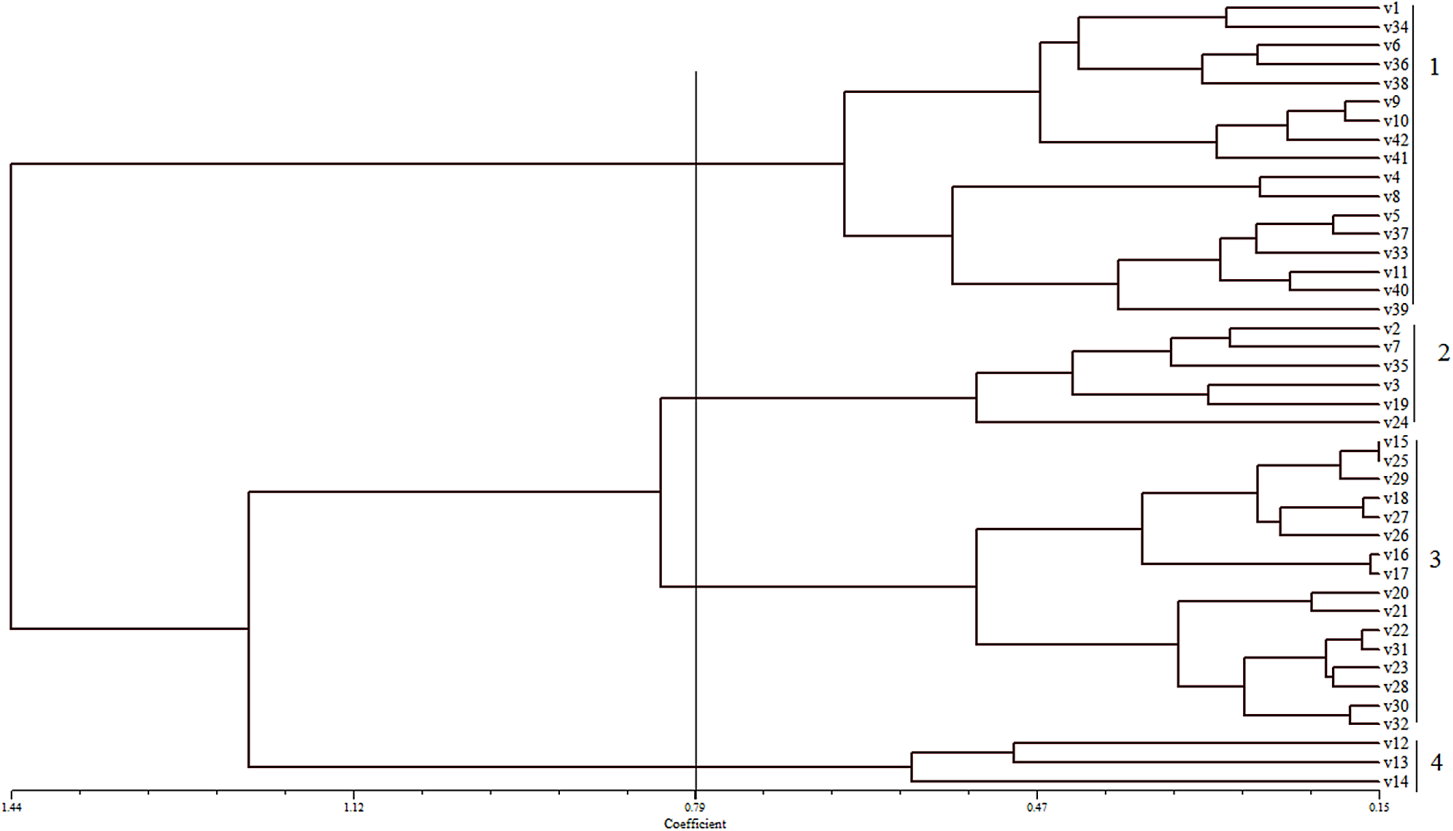
Dendrogram constructed from morphological and agronomic data.

The similarity coefficient as shown in the dendrogram varied from 0.15 to 1.44 (Fig 4). All the genotypes were grouped into 4 groups (Fig 4) at 0.79 coefficient. Group1 comprises of red rice genotypes from several geographical origins. This group had the highest mean value of agro-morphological traits, such as plant height (148.78 cm), length of flag leaf (53.18 cm), percentage of filled grain (66.45%), panicle length (25.46%), kernel length (6.05 mm), and harvest index (0.77). Three genotypes were found in group 4, and this cluster had the lowest mean for traits such as number of tillers (2.04), number of panicles (1.55), percentage filled grain (31.51%), harvest index (0.09), grain yield/plant (0.65g), kernel length (5.59 mm), and kernel length/breadth ratio (3.19).

### Principal component analysis from agro-morphological traits data

**Fig 5:**
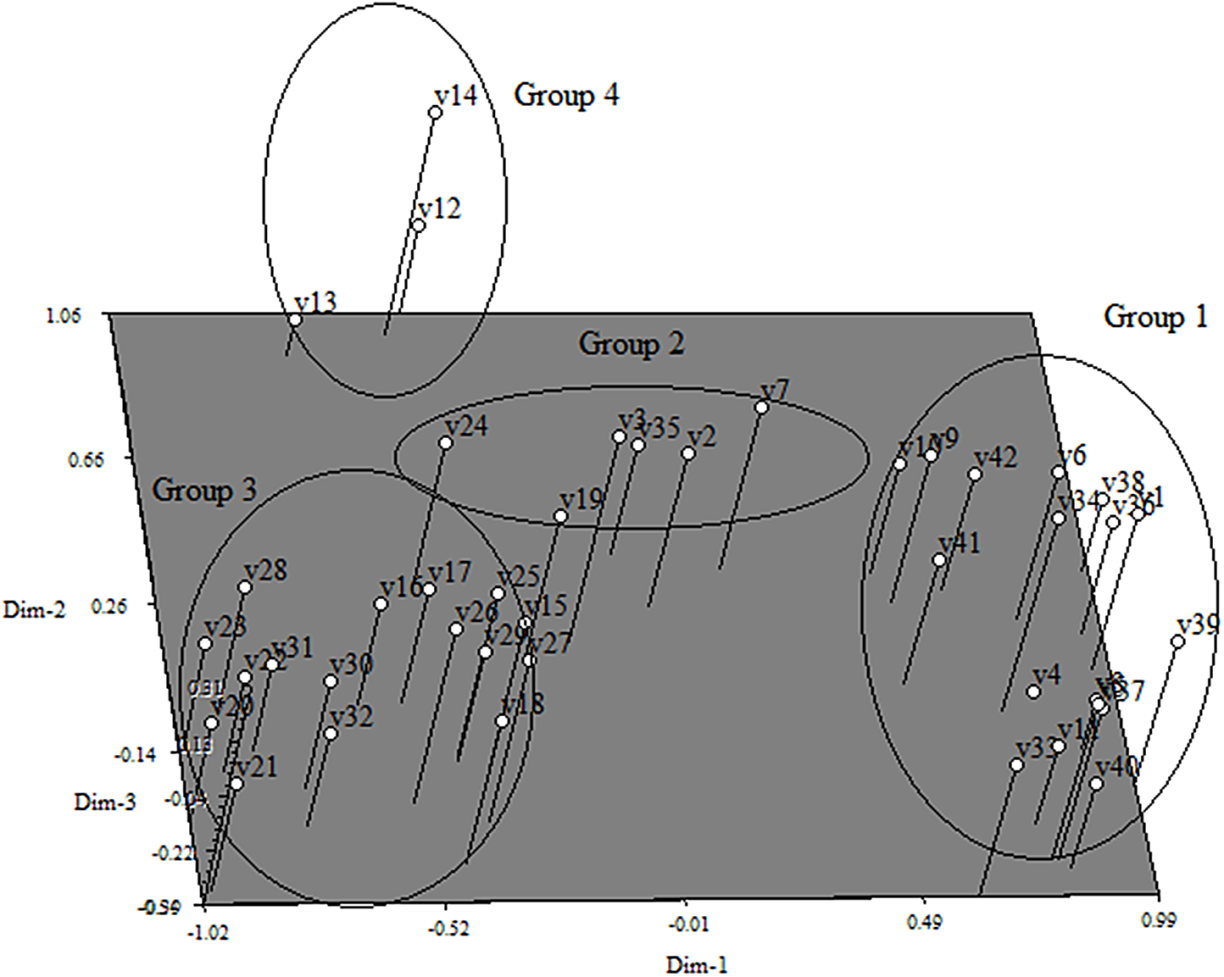
Principle component analysis (PCA) from morphological and agronomic data.

Using the PCA all the rice genotypes were grouped into 4 distinct groups which were similar to the cluster analysis grouping (Fig 5). All agro-morphological traits showed the highest cumulative percentage (≥70%) except for length breath ratio (69%), chlorophyll SPAD value at 40 days (58%) and 60 days (56%) (Table 6).

**Table 6:** Principle component analysis (PCA) explanation for 5 components of agro-morphological traits.

Morphological and agronomical traits	pc1	pc2	pc3	pc4	pc5	Final communality	Percentage
**Days flowering**	0.91	0.09	0.02	0.06	0.07	0.85	85
**Days maturity**	0.89	-0.07	-0.16	0.15	0.09	0.86	86
**Harvest index**	-0.63	0.33	0.54	0.24	0.03	0.85	85
**Length flag leaf**	-0.76	-0.24	-0.004	-0.04	0.32	0.74	74
**Plant height**	-0.79	-0.36	-0.07	-0.006	0.26	0.82	82
**Panicle number**	0.03	0.94	0.14	0.07	0.09	0.91	91
**Tiller number**	0.17	0.9	0.04	0.13	0.1	0.87	87
**Panicle length**	-0.27	-0.63	-0.21	0.22	0.32	0.67	67
**Chlorophyll content 60 days**	-0.02	-0.01	0.75	-0.02	0.01	0.56	56
**100 grain weight**	-0.2	0.35	0.65	-0.26	0.31	0.75	75
**Yield/plant**	-0.27	0.42	0.56	0.39	0.3	0.8	80
**Chlorophyll content 40 days**	0.27	0.12	0.55	-0.16	-0.4	0.58	58
**% filled grain**	0.02	0.19	0.1	0.91	-0.12	0.89	89
**Kernel length**	0.03	-0.02	0.2	-0.27	0.82	0.78	78
**L/B ratio**	-0.1	0.1	-0.06	-0.07	0.81	0.69	69
**Eigenvalue**	3.54	2.79	2.12	2.07	2.04		
**Variation (%)**	28.18	22.21	16.88	16.48	16.24		
**Cumulative (%)**	28.18	50.4	67.28	83.76	100		

### Correlation among the agro-morphological traits data

Yield per plant had positive correlation with number of tillers, number of panicles, percentage filled and unfilled grain, harvest index, panicle length, and chlorophyll SPAD reading at 60 days (Table 7). Chlorophyll SPAD reading at 40 days showed significant negative assosication with plant height, flag leaf and panicle lenght.

**Table 7:** Pearson’s correlation coefficient among 15 agro-morphological traits of 42 coloured upland rice germplasms.

	No tiller	No panicle	Plant height	Flag leaf	% filled grain	100 gw	Hi	Pl	Y/P	Df	Dm	Klength	L/b ratio	Chloro 40	Chloro 60
**No tiller**	1														
**No panicle**	0.95**	1													
**Plant height**	(-0.47)**	(-0.41)**	1												
**Lgth flag leaf**	(-0.30)*	(-0.23)ns	0.89**	1											
**%filled grain**	0.40**	0.55**	(-0.06)ns	(-0.004)ns	1										
**100 gw**	(-0.07)ns	(-0.08)ns	(-0.03)ns	(-0.02)ns	(-0.19)ns	1									
**Hi**	0.18 ns	0.39*	0.27 ns	0.34*	0.71**	0.06 ns	1								
**Pl**	(-0.29) ns	(-0.25) ns	0.73**	0.80**	(-0.09)ns	0.16 ns	0.29 ns	1							
**Y/p**	0.39 **	0.55**	0.10 ns	0.19 ns	0.68**	0.08 ns	0.78**	0.34*	1						
**Df**	0.27 ns	0.10 ns	(-0.60)**	(-0.60)**	(-0.17)ns	0.06 ns	(-0.50)**	(-0.30)ns	(-0.19) ns	1					
**Dm**	0.17 ns	(-0.0009)ns	(-0.56)**	(-0.59)**	(-0.23)ns	0.08 ns	(-0.54)**	(-0.29)ns	(-0.25) ns	0.98**	1				
**Klength**	0.03 ns	0.03 ns	0.14 ns	0.25 ns	(-0.08) ns	0.59**	0.10 ns	0.33*	0.15 ns	0.02 ns	(-0.04) ns	1			
**L/b ratio**	0.15 ns	0.13 ns	0.18 ns	0.26 ns	0.02 ns	(-0.05) ns	0.09 ns	0.34*	0.19 ns	(-0.07) ns	(-0.14) ns	0.63**	1		
**Chloro 40**	0.23 ns	0.26 ns	(-0.51)**	(-0.42)**	0.03 ns	(-0.05)ns	0.09 ns	(-0.44)**	0.05 ns	0.11 ns	0.10 ns	(-0.11) ns	(-0.31)*	1	
**Chloro 60**	0.18 ns	0.22 ns	(-0.04) ns	0.12 ns	0.39*	(-0.0004) ns	0.38*	0.05 ns	0.41**	(-0.12) ns	(-0.22) ns	0.10 ns	0.09 ns	0.30*	1

No tiller- number of tiller, No panicle-number of panicle, 100 gw -100 grain weight, Hi-harvest index, Pl-panicle length, Y/P-yield per plant, Df-days to flowering, Dm-days to maturity, Klength-kernel length, L/b ratio-length breadth ratio, Chloro 40- chlorophyll SPAD reading at 40 days after planting, Chloro 60- chlorophyll SPAD reading at 60 days after planting

*- significant at 0.05 level

**- significant at 0.01 level, ns- not significant

### Genetic parameters

The genetic parameters calculated, such as genotypic variance (σ^2^g), phenotypic variance (σ^2^p), heritability (h^2^_B_), and genetic advance (GA), are presented in Table 8. The broad sense heritability for 15 agro-morphological traits ranged from 17.68% (chlorophyll SPAD value at 40 days) to 99.69% (kernel length), respectively. Yield per plant showed the highest value of genetic advance (126%) among all the traits. Broad sense heritability was high for virtually all the yield component traits with the lowest (percentage filled grain) being approximately 62%.

**Table 8:** Genetic parameter calculated among 15 agro-morphological traits.

haracter	Mean	MSG	MSE	σ^2^g	σ^2^p	PCV	GCV	h_β_ (%)	GA(%)
**NT**	2.67	0.99	0.18	0.27	0.45	25.12	19.46	60	30.90
**NP**	2.59	0.98	0.2	0.26	0.46	26.19	19.69	56.52	30.53
**PH**	124.73	2112.97	37.17	691.93	729.1	21.65	21.09	94.9	43.05
**LF**	43.79	354.61	31.39	107.74	139.13	26.94	23.7	77.44	42.83
**PL**	23.34	65.95	2.23	21.24	23.47	20.76	19.75	90.5	39.37
**%FG**	67.59	211.34	36.29	58.35	94.64	14.39	11.3	61.65	17.89
**100GW**	2.32	1.47	0.008	0.49	0.5	30.48	30.17	98	38.32
**HI**	0.66	0.19	0.01	0.06	0.07	40.09	37.11	85.71	70.86
**Y/P**	3.54	5.74	0.56	5.18	5.74	67.68	64.29	90.24	126
**Dflower**	80.64	573.26	2.55	190.24	192.79	17.22	17.1	98.68	34.67
**Dmature**	145.15	744.49	1.73	247.57	249.3	10.88	10.84	99.31	22.43
**Klength**	6	0.97	0.001	0.32	0.321	9.44	9.43	99.69	18.48
**Lbratio**	3.42	0.42	0.002	0.15	0.152	11.4	11.32	98.68	22.37
**Chloro40**	38.17	18.69	11.36	2.44	13.8	9.73	4.09	17.68	3.71
**Chloro60**	39	14.49	5.53	2.99	8.52	7.48	4.43	35.09	5.05

NT-number of tiller, NP-number of panicle, PH-plant height, LF-length of flag leaf, PL-panicle length, %FG-percentage filled grain, % UFG- percentage unfilled grain, 100GW- 100 grain weight, HI- harvest index, Y/P- yield per plant, d flower- days to flowering, d mature- days to maturity, Klength-kernel length, Lbratio-length breadth ratio, Chloro40- chlorophyll SPAD reading at 40 days, Chloro 60- chlorophyll SPAD reading at 60 days, MSG- mean square germplasms, MSE-mean square error, σ^2^g- genotypic variance, σ^2^p- phenotypic variance, PCV- phenotypic coefficient of variance, GCV-genotypic coefficient of variance, h_β_ (%)-broad sense heritability, GA(%)-genetic advance.

## Discussion

Genetic divergence as revealed by the molecular markers and agro-morphological traits is important for breeding and improvement of existing rice genotypes to suit consumer demands. Genetic divergence helps in breeding resistance and tolerance to various biotic and environmental stresses and also as a tool to investigate the effects of climate change, in order to select the genotypes with higher potential for use in breeding programmes. Among the 25 SSR primers used in this experiment, only 21 show polymorphism among genotypes. The marker used must be informative in order to reveal the genetic divergence among the genotypes. The mean polymorphic information content (PIC) recorded was 0.41 for all the SSR markers tested. The marker is informative if the PIC value is higher than 0.5 [21]. For instance, Ravi et al. [22] reported the mean PIC value of 0.578 for genetic diversity analysis of rice cultivars using SSR data.

Cluster analysis of SSR primers data grouped all the genotypes based on geographical origin and status of the genotypes. Additionally, only one genotype was placed in Group 5. This genotype was an advanced cultivar from the Ivory Coast. The cophenatic correlation value (r) calculated from the dendrogram was 0.83 which shows the good fit of the data for diversity analysis if (r>0.8). Based on the grouping in the PCA, some groups could not be separated using clustering analysis. Groups 3 and 5 (Fig 2), for example, consisted of mixed groupings of genotypes and this cannot be resolved by PCA. Thus, this analysis can be informative for differentiation among the major groups and it will help the breeder to select from diverse breeding lines.

All agro-morphological traits differed significantly. Three genotypes showed the lowest values for yield per plant and percentage of filled grain. The breeding line showed good agro-morphological traits, such as lower plant height, high filled grain, and higher grain yield per plant compared with the landrace or traditional cultivars. Thus, cross breeding between traditional cultivars and breeding lines can be done to produce plants with good yield characteristics.

The dendrogram for agro-morphological traits was constructed based on the matrix of average of taxonomic distance using the UPGMA method. The dendrogram showed that Group 1 consisted of all the landrace and traditional cultivars. These genotypes displayed similar agro-morphological characteristics, such as plant height, panicle length and length of flag leaf. Group 3 showed higher mean value of grain yield per plant (3.9g) as it was from the breeding lines from Philippines.

From the dendrogram constructed based on SSR marker and agro-morphological traits, there are differences in grouping of the genotypes. Agro-morphological traits were influenced by environmental factors such as light intensity, disease and humidity. Additionally, clustering based on SSR markers was more accurate. This is because their usage is not influenced by environmental factor thus it will reflect the actual level of genetic difference existing among the genotypes [23]. In addition, SSR markers can detect slight differences of DNA structure.

From PCA analysis, the first four components explained about 83.76% of variation [24]. Since the variation is high (≥ 25%), this analysis can be used along with cluster analysis to show the relatedness among the genotypes [25]. From the results of the first three components, it was seen that days to first flowering, number of panicles, number of tillers and percentage of filled grain played an important role in explaining the variation. This is because positive eigenvalue is shown for each of the 3 components.

Plant height showed a negative correlation with number of tillers and number of panicles. Thus, lower plant height is a good characteristic because it results to higher tiller and panicle numbers. From the result (Table 5), traditional varieties showed the highest plant height when compared with breeding line. In modern rice breeding, gene *sd*-1 is one of the most important genes controlling dwarfisim in rice plant. It ressesive character helps to improve lodging resistance as a result of shortened culm [26]. Lodging is the most common problem affecting upland rice in the field. Thus reducing the plant height is one of the means of resolving the lodging problem. Most of the modern rice varieties have short plant height and this is the focus of selection by breeders for improvement of rice plant. Thus, plant height is one of the most important traits which needs to be considered in production of high yield variety. In addition, yield per plant was positively correlated with number of tillers, number of panicles, percentage of filled grain, harvest index and panicle length. This information is valuable to know the agro-morphological traits that contribute to the to yield of the plant.

From the present study, higher phenotypic variance was recorded than genotypic variance for all traits evaluated. The difference in value of genotypic variance and phenotypic variance was due to environmental factor. Our data shows that environmental factors have pronounced effect on each of those traits compared to the genotypic factors. Broad sense heritability was found to be high for plant height, panicle length, 100 grain weight, harvest index, yield per plant, days to flowering, days to maturity, kernel length and length breadth ratio. The heritability results must be combined with expected genetic advance to achieve reliable results [27]. Yield per plant and harvest index showed the highest value for both heritability and genetic advance. These traits need to be considered for yield improvement of rice plant genotypes through breeding programmes. High heritability and genetic advance for grain yield per plant and harvest index has also been reported by Bisne et al. [28].

From the analysis it is shown that all the 42 coloured upland rice genotypes can be selected for breeding programmes based on the SSR primers and agro-morphological traits evaluation. Genotypes such as C, Chirikata 2, Ble Chu Cau, and IR 9669-PP 836–1 have high potentials for selection. This is because, these genotypes have good agro-morphological traits such as, high percentage of filled grain, high harvest index and high grain yield per plant. Furthermore, the selected genotypes are clustered in different groups based on the SSR marker analysis which indicates that they are genetically divergent and thus breeding from these genotypes can produce good progenies with superior traits.

## Conclusions

Cluster analysis from the SSR markers grouped all the genotypes into 7 groups according to geographical origin and status of the cultivar. All agro-morphological data showed significant differences at p≤0.05 and p≤0.01 for all traits which shows the presence of diversification among the 42 coloured upland rice genotypes. Four groups were constructed using agro-morphological data clustering analysis. Yield contribution factors traits, which are yield per plant and harvest index, showed the highest value of heritability and genetic advance. Selection based on these traits can be done for further breeding programmes. Potential genotypes such as C, Chirikata 2, Ble Chu Cau, and IR 9669-PP 836–1, are recommended for selection and further evaluation in future breeding programmes. This is based on their molecular and agro-morphological information. These genotypes, selected from groups 2, 3, 4 and 5 from the SSR primer grouping, also have high grain per yield and harvest index values. For further studies, the evaluation of nutritional value of coloured upland rice can be done since red and purple bran may contain many phytochemical and neutraceutical functional foods. Thus, it will give high impact on rice breeding programme for development high yield and functional rice.

## Abbreviations

CTAB: Cetyltrimethyl ammonium bromide
DNA: Deoxyribonucleic acid
EDTA: Ethylene diaminetetraacetate
GA: Genetic advance
He: Heterozygosity
mM: Millimolar
IRRI: International rice research institute
MOP: Murate of potash
TSP: Triple super phosphate
NaCl: Sodium chloride
PVP: Polyvinylpolypyrrolidone
SAHN: Sequential agglomerative hierarchal nested
SDS: Sodium dodecyl sulphate
TE: Tris-EDTA
μL: Microliter
